# Survival analysis after head injury: is a normal INR reassuring?

**DOI:** 10.1186/cc12274

**Published:** 2013-03-19

**Authors:** K Leclerc-Gagne

**Affiliations:** 1 Hôpital Sacré-Coeur de Montréal, Canada

## Introduction

Intracranial bleeding after head injury is an important issue in the emergency department (ED), and elevated INR increases this risk [1]. A retrospective study showed an association between different levels of normal INRs (<1.6) and elevated risk of intracranial bleeding, with a significant elevation of this risk over an INR threshold of 1.3 [2]. The objective of the study was to evaluate clinical impact of an INR within the normal range in patients with head trauma. We compared mortality between patients with INR <1.3 and those with INR ≥1.3 to <1.6.

## Methods

A *post hoc *analysis of prospective data collected from 3,356 patients seen in a tertiary-care ED from March 2008 to February 2011. We included patients aged 16 years old and over with an INR <1.6 and a head CT performed within 24 hours of the trauma. We followed these patients until December 2012, performed a chi-square test between mortality of the two groups and calculated the hazard ratio (HR) from survival analysis using Cox regression.

## Results

**Figure 1:**
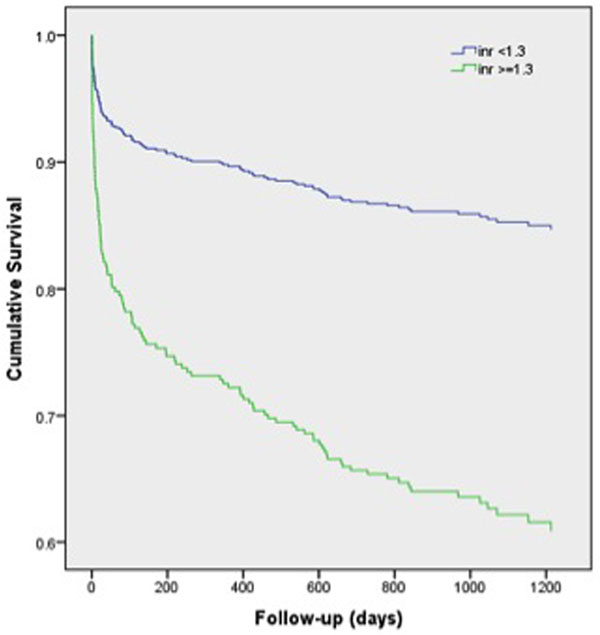
**Survival analysis of patients with INR <1.3 and with INR ≥1.3**.

Patient mean age was 55.1 years (SD ±23), 65% were men and mean follow-up duration was 3.1 years (SD ±0.8). A total of 115 patients (15.9%) died during follow-up: 16 (36.4%) in the group with INR ≥1.3 and 99 (14.6%) in the group with INR <1.3 (*P <*0.001). Results showed a significantly higher risk of death in the group with INR ≥1.3: HR = 2.99 (95% CI = 1.8 to 5.1); *P <*0.001 (Figure 1).

## Conclusion

In patients with head injury and normal INR (<1.6), there is an association between an INR ≥1.3 and higher risk of death. Therefore, it would be useful to request an INR in patients presenting with a head injury when bleeding is suspected, even in the absence of anticoagulant.
